# Molecular diversity of *Paenibacillus larvae* strains isolated from *Lithuanian apiaries*

**DOI:** 10.3389/fvets.2022.959636

**Published:** 2022-08-22

**Authors:** Paulina Amšiejute, Vaclovas Jurgelevičius, Petras Mačiulskis, Ceslova Butrimaite-Ambrozevičiene, Simona Pilevičiene, Zygimantas Janeliunas, Tatjana Kutyriova, Ingrida Jacevičiene, Algimantas Paulauskas

**Affiliations:** ^1^National Food and Veterinary Risk Assessment Institute, Vilnius, Lithuania; ^2^Faculty of Natural Sciences, Vytautas Magnus University, Kaunas, Lithuania

**Keywords:** American foulbrood (AFB), ERIC I, honey bee (*Apis mellifera*), MLVA, *Paenibacillus larvae*

## Abstract

*Paenibacillus larvae* bacterium is known to be the causative agent of American foulbrood (AFB), a widespread, highly contagious and fatal disease in honey bees (*Apis mellifera*). There are four genotypes of *Paenibacillus larvae* that are named after their enterobacterial repetitive consensus (ERIC), and a fifth ERIC genotype has recently been found. In this study, a total of 108 independent *P. larvae* isolates from different geographical regions in Lithuania collected between 2011 and 2021 were investigated by molecular methods. The aims of this study were to detect which enterobacterial repetitive intergenic consensus (ERIC) genotype is the most common in Lithuania apiaries, identify and differentiate subtypes of the defined genotype by using multiple-locus variable number of tandem-repeat analysis (MLVA), and review how bacterial molecular diversity has changed over time in different parts of Lithuania. The obtained molecular analysis results showed that 100% of *P. larvae* bacterial isolates from Lithuania belong to the ERIC I genotype and can be differentiated to nine different subtypes by using the MLVA and capillary electrophoresis methods.

## Introduction

*Paenibacillus larvae* (*P. larvae)* is dangerous and recognized worldwide to be the causative agent of American foulbrood (AFB), a serious and fatal bacterial disease that affects the brood (below 36 h of age) of honey bees (mostly *Apis mellifera*) (1, 2). *P. larvae* is a gram-positive, spore-forming bacterium (3) which spores can remain viable for several decades, can survive even in the most extreme conditions and are resistant to heat and antibiotics (1). AFB causes considerable losses of honey bee colonies and huge economic losses in the global apiary industry (1). *Paenibacillus larvae* can be easily spread when highly resistant spores are transmitted by swarming, drifting and foraging bees. Beekeeping routines, as example exchange of hive equipment and materials between colonies or apiaries can also increase and spread bacterial infection. An infected colony can have visible AFB clinical symptoms or be asymptomatic yet still infected (24). The primary symptoms by which AFB is identified are the brownish, sticky and partly fluid bodies of dead larvae (4).

The *P. larvae* genome contains preserved repetitive DNA sequences that vary in number and length within a species (5). Using enterobacterial repetitive intergenic consensus (ERIC)-based PCR (ERIC-PCR), *P. larvae* has been classified into four ERIC genotypes (ERIC I-IV), with a novel ERIC V genotype recently identified and described (3, 6). All ERIC genotypes differ in their biochemical, morphological and virulence characteristics. ERIC genotypes I and II have a worldwide distribution and are epidemiologically the most important types (7, 8), while genotypes ERIC III and IV have not been identified anywhere for several years and only a few examples can be found in culture collections (1). ERIC I and ERIC II have been detected in Germany, Finland, Sweden, Austria, Italy, and Republic of Kosovo (9, 22), ERIC I also has been found among Argentinian and Uruguayan *P. larvae* strains (22).

Over the years several genotyping methods have been applied and improved for studying the genetic diversity of *P. larvae*, including the repetitive element PCR fingerprinting (rep-PCR) with primers (10) beyond ERIC also BOX, MBO, and REP1 (11, 12), pulsed-field gel electrophoresis (PFGE) (3), restriction endonuclease fragment patterns (REFP), amplified fragment length polymorphism (AFLP), denaturing gradient gel electrophoresis (DGGE) (13) (25) and multilocus sequence typing (MLST) (14). An alternative method to MLST has recently been developed and adapted for genotyping of *P. larvae* by researchers in Belgium (2). The method, which is called multi-locus variable number of tandem-repeat analysis (MLVA) is based on different genome loci of various bacterial strains that have a variable number of tandem repeats (VNTR) caused by slipped strand mispairing. According to the research in Belgium, MLVA method is useful for epidemiological, phylogenetic relationship and evolutionary studies among *P. larvae* strains (2).

In Lithuania during the last 10 years, AFB and *P. larvae* spores have been detected in bees, bee broods, honey samples, wax samples and hive debris in bacteriological tests and using molecular methods (PCR). Scientific research was carried out under the “Support to the Lithuanian beekeeping sector” program in National Food and Veterinary Risk Assessment Institute of Lithuania (unpublished data). The prevalence of AFB infection varied in different years between 4.5 and 100%. No detailed information has been obtained on the occurrence of *P. larvae* strains in Lithuania. Therefore, this study was designed to confirm the presence, genotype and subtype characterization, and distribution of AFB in honey bee apiaries in Lithuania through the last 10 years of surveillance by using the MLVA method.

## Materials and methods

### Bacterial isolates

A total of 108 *P. larvae* field isolates were collected from ten regions of Lithuania and were provided by the National Food and Veterinary Risk Assessment Institute of Lithuania for genotyping. The isolates were collected from honey bee brood combs with and without AFB clinical symptoms between 2011 and 2021 as part of the AFB surveillance programme. The reference strain of *P. larvae* ERIC I (ATCC 9545) was used as the control for genotyping. Pure culture isolates of *P. larvae* were revived from frozen stocks (−70°C) by streaking onto blood agar plates followed by incubation at 37°C for 3–7 days under aerobic conditions. All purified isolates were confirmed as *P. larvae* by matrix-assisted laser desorption/ionization time-of-flight mass spectrometry (MALDI-TOF MS; Bruker Daltonik GmbH, Germany), and the phenotypic characterization of *P. larvae* ERIC genotype was performed by using the following biochemical tests: haemolysis [Columbia agar with 5% sheep blood media (BioMerieux)], production of bacterial pigment (MYPGP agar prepared in laboratory), catalase activity, oxidase activity, mobility (SIM agar), mobility in a liquid medium (nutrient broth) (OXOID, England). Fermentation of glucose, sucrose, xylose, lactose, maltose, arabinose, mannitol, trehalose, and salicin (SIGMA) was carried out by using phenol red broth base (Biolab, Hungary), 5 g/l agar (Liofilchem, Italy) and particular carb. Additional tests included Methyl red test and Voges-Proskauer reaction were made in Clark broth (Biomaxima, Poland). For phenotypic characterization also were used hydrogen sulfide (H_2_S) production, indole production, hydrolysis of urea and esculin, use of citrate (OXOID, England) and nitrate reduction (Liofilchem, Italy) tests (15, 23).

### DNA extraction

Material from pure isolated and confirmed *P. larvae* colonies was scraped off the agar plate and re-suspended in 200 μl sterile, deionised water and centrifuged for 2 min at 10,000 rpm. Supernatant was removed and the sediment re-suspended in 150 μl of 6% Chelex (Bio-Rad) solution. Tubes with samples were incubated at 56°C for 20 min and 250 rpm. After incubation, the tubes were vortexed at high speed for 10 s and heated at 99°C for 20 min and 250 rpm. In the last step, the tubes were again vortexed at a high speed for 10 s and centrifuged for 8 min at 12,000 rpm. The supernatant was then used as the DNA template for PCR. The concentration and purity of the DNA extracts were determined by DNA/RNA spectrophotometer (Nano Photometer P-Class, IMPLEN) at A260 and A280 wavelengths. DNA templates were diluted to 80–100 ng/μl. Concentrated and diluted DNA extracts were stored at −20°C until the start of the experiments.

### ERIC-PCR

First, ERIC typing was performed to identify the *P. larvae* genotype of the isolates. The PCR was carried out in a 25-μL tube volume, and the reaction was optimized to contain 1 × Multiplex PCR Master Mix (Qiagen, Hilden, Germany), 0.2 μM of each primer PLaERIC1for (5′-ATGTAAGCTCCTGGGGATTCAC-3′), PLaERICrev (5′-AAGTAAGTGACTGGGGTGAGCG-3′) and 5 μL of the DNA template (10, 12, 16). DNA from the reference strain (ATCC 9545) served as the positive control in the PCR trials and sterile distilled water as the negative control. The reaction conditions were as follows: 95°C for 15 min, 35 cycles at 94°C for 1 min, 53°C for 1 min and 72°C for 2.5 min, and a final extension step at 72°C for 10 min. To determine ERIC patterns, amplicons were analyzed by the QIAxcel Advanced System (Qiagen, Hilden, Germany), capillary electrophoresis using the QIAxcel DNA High-Resolution Kit, QX Alignment Marker 50–5,000 bp, QX Size Marker 100 bp-2.5 kb, OM500 separation method and a sample injection time of 10 s.

### Multiplex PCR and VNTR amplification

Molecular typing for ERIC subtype determination was performed using primers described by Descamps et al. (2) as follows: PLaVNTR A for (5′-GAGGGATATACCCCACCTCTTT-3′), PLaVNTR A rev (5′-GGGGAAGTATGATCCCGAAG-3′), PLaVNTR B for (5′-CCG GAA TAA TCC GCT TAT GA-3′), PLaVNTR B rev (5′-ATC ACC AGA GTT GGC GAT TC-3′), PLaVNTR C for (5′-TGG TTT AGG AAC CGG TGT TG-3′), PLaVNTR C rev (5′-CAC ATT AAA GCC TGT GCA GGT A-3′), PLaVNTR D for (5′-ATC ATG GCG GTT GGG ATG-3′), PLaVNTR D rev (5′-CAC AGG CTC GAC AAC CAC TA-3′), PLaVNTR E for (5′-TGT TCA ATT TTG ATT GTT TTG TTC A-3′) and PLaVNTR E rev (5′-TAT ATG GCG GTC GGC TTA AT-3′). Multiplex PCR was carried out in a final volume of 25 μl containing 1 × Multiplex PCR Master Mix (Qiagen, Hilden, Germany), five pairs of primers representing five different loci (VNTR A, VNTR B, VNTR C, VNTR D and VNTR E) and 5 μl of DNA template. The primers' concentration and reaction conditions with slight modifications were used according to Descamps et al. (2). The reaction conditions were as follows: 95°C for 10 min, 30 cycles at 94°C for 1 min, 52°C for 1 min and 72°C for 1 min, and a final extension step at 72°C for 10 min. Amplification products were analyzed by the capillary electrophoresis QIAxcel Advanced System (Qiagen, Hilden, Germany) using the QIAxcel DNA High-Resolution Kit, QX Alignment Marker 15–3,000 bp, QX Size Marker 50–800 bp, and QX Alignment Marker 15–1,000 bp, QX Size Marker 50–500 bp, OM500 separation method and a sample injection time of 10 s. The Biocalculator QIAxcel software also sized the fragment length and produced a virtual gel image for each run.

### Data analysis

Visualization of the geographic location of each apiary was used to show the spatial distribution of the *P. larvae* isolates obtained and the collection date to show their temporal distribution. The obtained data were visualized using a web application “Microreact” (17).

## Results

Biochemical analysis of the bacterial cultures showed that all *P. larvae* isolates belonged to the ERIC I genotype (8, 15, 23). Preliminary *P. larvae* genotype results were confirmed by the molecular ERIC-PCR method (3) after capillary electrophoresis data analysis. Typical patterns for ERIC II, III, IV, and V were not detected by the ERIC-PCR method. More comprehensive ERIC I genotype analysis was undertaken using multiplex PCR primers (VNTR A, VNTR B, VNTR C, VNTR D, VNTR E) and the MLVA method. Isolates were differentiated according to their VNTR lengths (Supplementary Table 1) using the QIAxcel Advanced system capillary electrophoresis (Supplementary Table 2). MLVA method conditions with minor modifications for *P. larvae* strain analysis and study results interpretation were done according to Descamps et al. (2).

**Figure 1:**
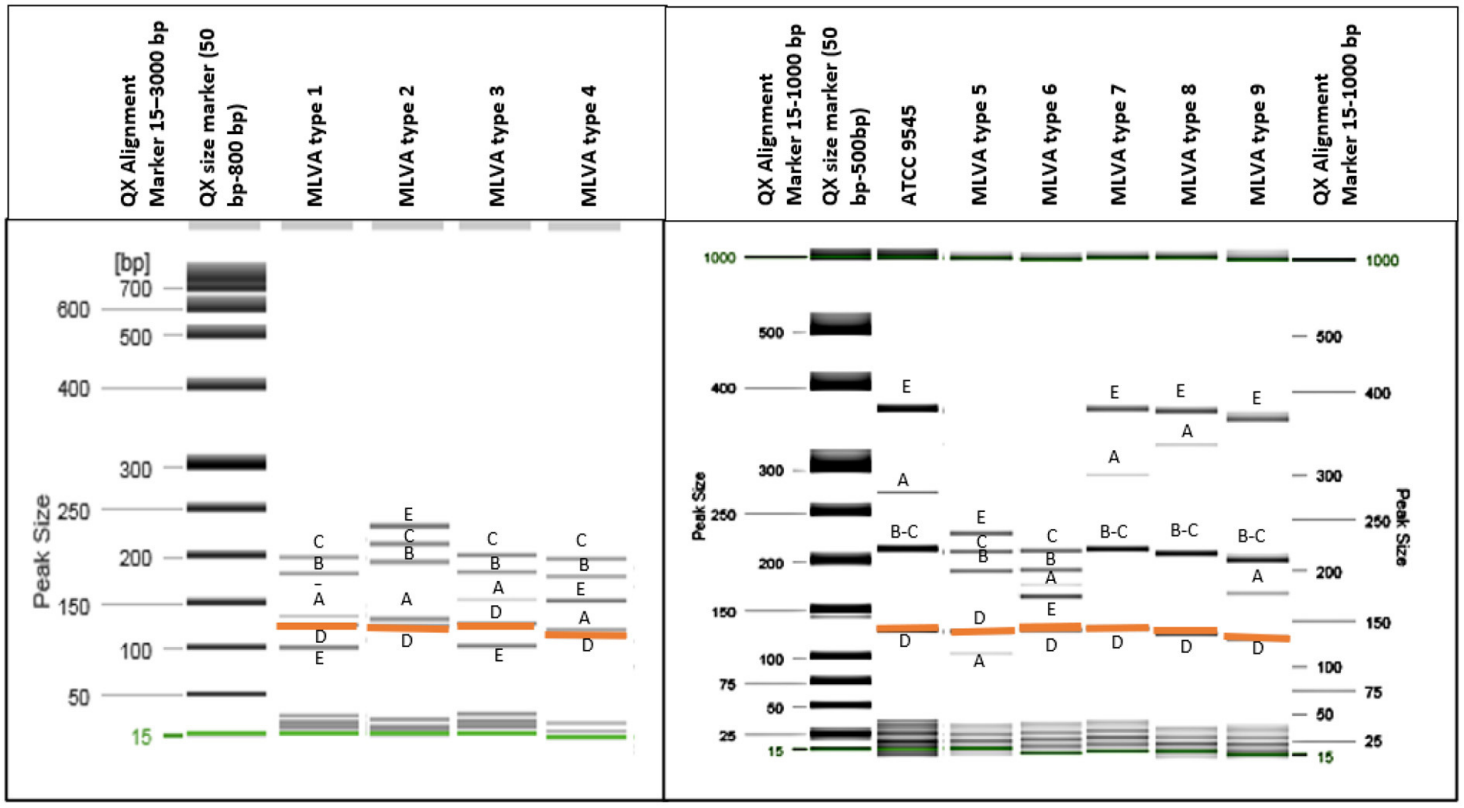
Nine different MLVA types of *P. larvae* isolates were identified using QIAxcel capillary electrophoresis (gel image) method. All isolates had a band of around 125 bp length [orange color **(D)**], and all five loci were amplified. **(A–E)** Indicate each VNTR locus. Two bands which differ in very small bp number and are not separated clearly [loci **(B,C)**] have more intense dark color.

All analyzed *P. larvae* bacterial strains had the 125 bp band (Figure 1) of the VNTR D locus and all VNTR loci (VNTR A, VNTR B, VNTR C, VNTR D, VNTR E) were amplified, resulting in all bacterial strains being clustered in the ERIC I genotype (2). Using the MLVA method and five different VNTR loci, nine different MLVA patterns could be detected (Figure 1; Table 1). Also, the variability between same type MLVA patterns were noticed (Supplementary Table 3; Supplementary Figure 1). VNTR A and VNTR E loci were found to be the most diverse compared with the others. Therefore, the bacterial strains were differentiated into nine types based on the allele lengths of these loci (Figure 1; Table 1). The most common in Lithuania was the first MLVA type, with a total of 48 (44.44%) isolates assigned to this type. The prevalence of MLVA types 2 and 3 patterns was 34.26 and 12.96%, respectively. Other MLVA types were very rare and had only one or two representative isolates (Table 1). None of the isolates matched the *P. larvae* certified reference culture (ATCC 9545) MLVA type. Only types 7, 8, and 9 were similar, with the main difference being the number of tandem repeats of the locus VNTR A allele. Also, these MLVA types after several test repeats showed amplification of longer band of VNTR B. Three isolates of brood samples with typical signs of AFB were clustered in MLVA type 2 and one clustered in MLVA type 1. Selected isolates of different MLVA patterns were amplified by multiplex PCR a few times and, using QIAxcel, gave reproducible results. Representative type isolates were also amplified using each VNTR loci primer separately for more accurate identification of the target allele length.

**Table 1:** Prevalence of different ERIC I subtypes distinguished by MLVA analysis.

**MLVA types**	**VNTR A TR**	**VNTR B TR**	**VNTR C TR**	**VNTR D TR**	**VNTR E TR**	**Number of isolates**	**Prevalence (%)**
1	3/4^a^	6	3/4^c^	3	0	48	44.44
2	3/4^a^	6	3/4^c^	3	1/2^d^	37	34.26
3	5/6^a^	6	3/4^c^	3	0	14	12.96
4	3/4^a^	6	3/4^c^	3	0/1^d^	2	1.85
5	2	6	3/4^c^	3	2	2	1.85
6	6	6	3/4^c^	3	1	2	1.85
7	12	6/7^b^	3/4^c^	3	4	1	0.93
8	15	6/7^b^	3/4^c^	3	4	1	0.93
9	6	6/7^b^	3/4^c^	3	4	1	0.93
ATCC 9545	11	6/7^b^	3/4^c^	3	4	1	

^a^*Number of tandem repeats (TR) varies between a small number of bp: 3 TR (121 bp) and 4 TR (140 bp), 5 TR (159 bp, and 6 TR (178 bp)*.

*^b^TR varies between a small number of bp: 6 TR (191 bp) and 7 TR (212 bp)*.

*^c^TR varies between a small number of bp: 3 TR (199 bp) and 4 TR (223 bp)*.

*^d^0 means that the amplifiers are about 100 bp length, 0/1–150 bp, 1/2 a little over 200 bp*.

**Figure 2:**
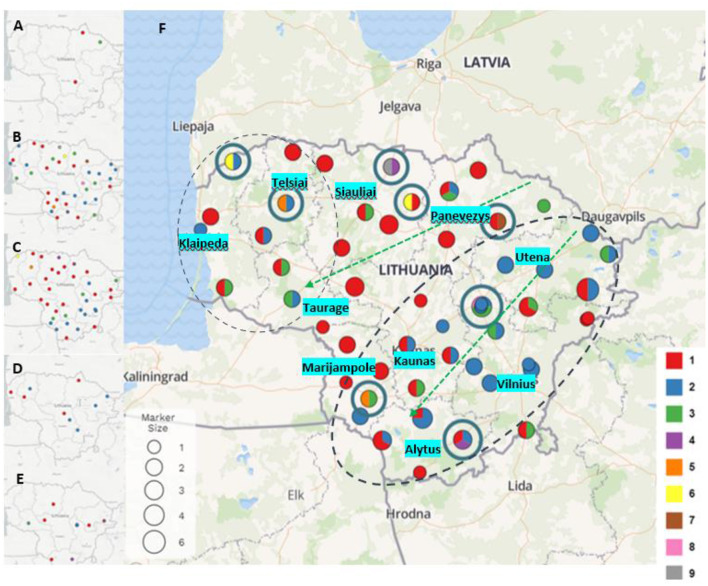
*Paenibacillus larvae* MLVA types detected in 2011 **(A)**, 2015 **(B)**, 2016 **(C)**, 2017 **(D)**, and 2021 **(E)** (type 1- red, type 2 - blue, type 3 - green, type 4 - purple, type 5 - orange, type 6 - yellow, type 7 - brown, type 8 - pink, type 9 - gray). Map **(F)** visualizes all *P. larvae* samples and all MLVA types collected and investigated over the years. Dot size indicates the number of samples collected in the same or nearby apiaries over the years (from 1 to 6). Dots with blue circles indicate the rarest MLVA types (types 4, 5, 6, 7, 8, and 9). Green arrows show distribution of MLVA type 3, and two black dash circles show distribution of MLVA type 2 over the time.

Based on obtained results, an analysis was undertaken of how different *P. larvae* MLVA types had spread geographically in different parts of Lithuania over the 10 years (Figure 2). In *P. larvae* samples collected in 2011, two MLVA types were identified: type 1 (*n* = 2) and type 3 (*n* = 1) (Figure 2A). Unfortunately, owing to the small number of samples (*n* = 3), the prevalence of the MLVA types in the country cannot be evaluated. Samples collected in 2015 (*n* = 41) were infected with largest variety of *P. larvae* MLVA types (Figure 2B). Totally eight different MLVA patterns were identified: type 1 (*n* = 14; 34.15%), type 2 (*n* = 15; 36.58%), type 3 (*n* = 7; 17.07%), type 5 (*n* = 1; 2.44%), type 6 (*n* = 1; 2.44%), type 7 (*n* = 1; 2.44%), type 8 (*n* = 1; 2.44%), and type 9 (*n* = 1; 2.44%). Six different MLVA types of *P. larvae* were identified in samples collected in 2016 (*n* = 40) (Figure 2C): type 1 (*n* = 22; 55%), type 2 (*n* = 11; 27.5%), type 3 (*n* = 4; 10%), type 4 (*n* = 1; 2.5%), type 5 (*n* = 1; 2.5%), and type 6 (*n* = 1; 2.5%). MLVA type 1 (*n* = 4; 44.44%) and type 2 (*n* = 5; 55.55%) were identified in samples collected in 2017 (*n* = 9) (Figure 2D), and in *P. larvae* samples collected in 2021 (*n* = 15) (Figure 2E), four MLVA types were identified: type 1 (*n* = 6; 40%), type 2 (*n* = 6; 40%), type 3 (*n* = 2; 13.33%), and type 4 (*n* = 1; 6.66%).

*P. larvae* samples (*n* = 108) were collected in ten regions of Lithuania between 2011 and 2021 (Figure 2). During the investigation period the largest variety of MLVA types were detected in Siauliai (*n* = 13) region: type 1 (*n* = 9), type 3 (*n* = 1), type 4 (*n* = 1), type 6 (*n* = 1), and type 9 (*n* = 1). Four different MLVA types were detected in Klaipeda (*n* = 7) region: type 1 (*n* = 3), type 2 (*n* = 2), type 3 (*n* = 1), and type 6 (*n* = 1), and in samples (*n* = 9) collected from Marijampole region: type 1 (*n* = 5), type 2 (*n* = 2), type 3 (*n* = 1), and type 5 (*n* = 1). In samples (*n* = 10) from Panevezys region were detected four different MLVA types as well: type 1 (*n* = 6), type 2 (*n* = 1), type 3 (*n* = 2), and novel type 7 (*n* = 1). Most common MLVA types [type 1 (*n* = 3), type 2 (*n* = 11), type 3 (*n* = 4)] and novel MLVA type 8 (*n* = 1) were also detected in brood samples (*n* = 19) collected in Vilnius region. Variety of three different MLVA types were detected in five regions of Lithuania: Alytus [type 1 (*n* = 5), type 2 (*n* = 5), and type 4 (*n* = 1)], Kaunas [type 1 (*n* = 7), type 2 (*n* = 3) and type 3 (*n* = 1)], Taurage [type 1 (*n* = 2), type (*n* = 1), and type 3 (*n* = 2)], Telsiai [type 1 (*n* = 3), type 2 (*n* = 2), and type 5 (*n* = 1)] and Utena [type 1 (*n* = 5), type 2 (*n* = 10), and type 3 (*n* = 2)].

MLVA type 1 analysis showed that this most common *P. larvae* genetic type first time was detected in north and southwest Lithuania, and over the years after collecting and testing more samples also have been detected in all regions of Lithuania. MLVA type 2 was detected for the first time in 2015 in apiaries located in the northwest and all along from the east to the southern part of Lithuania. In 2016, 2017, and 2021 a few more apiaries appeared with defined MLVA type 2. MLVA type 3 first time detected in 2011, and its geographic distribution showed that this type is most common for apiaries which are located from the northeast to the west areas and from the northeast to the southwest areas of Lithuania. Other MLVA types were detected in various parts of Lithuania: MLVA type 4 was identified in the 2016 and 2021 samples, MLVA types 5 and 6 were identified in the 2015 and 2016 samples, and MLVA types 7, 8, and 9 were identified in the 2015 samples (Figure 2).

## Discussion

This study is the first report of the genetic differentiation and distribution of *P. larvae* bacteria in Lithuania using the MLVA method and capillary electrophoresis system. The aims of the present study were to detect which enterobacterial repetitive intergenic consensus (ERIC) genotypes were most common in Lithuanian apiaries, identify and differentiate subtypes of the defined genotype, and analyse bacterial molecular diversity in different years and regions of Lithuania.

Sequencing is currently considered the gold standard method in bacterial identification and genotyping. However, due to the high cost and complexity of equipment, recent attempts have been made to find simpler but also sufficiently effective methods of genotyping. One of these is the multi-locus variable number of tandem-repeat analysis (MLVA). The molecular genotyping method based on MLVA was used for the first time for *P. larvae* genotyping by Descamps et al. (2). Furthermore, the new approach to variable number tandem repeat (VNTR) analysis using the QIAxcel capillary electrophoresis system was first reported in Japan in 2013 (18).

This study investigated 108 isolates from field samples collected between 2011 and 2021. Highest number of isolates were successfully revived from brood samples collected in 2015 (*n* = 41) and 2016 (*n* = 40). The present study identified ERIC I (100%) as the dominant ERIC genotype in Lithuania in the period from 2011 to 2021. It is an important result because ERIC I is known as less virulent than other common and widespread ERIC II genotype. According to other countries studies the ERIC I genotype was mostly detected in border regions close to Poland, Slovakia, Austria (22), and has also been observed in central Italy (19). Meanwhile, in most other investigations both ERIC I and ERIC II genotypes were observed. In Slovenia (16), Czech (22), and northern Italy (19) ERIC II was more frequent than ERIC I, except Republic of Kosovo (9) where ERIC I was more common genotype.

The MLVA method used in this study also revealed the existence of ERIC I subtypes that could not be distinguished using the conventional typing (ERIC-PCR) method. In this study, capillary electrophoresis was used for the MLVA and ERIC-PCR amplicons analysis. Using the MLVA method and VNTR analysis, nine different ERIC I subtypes were distinguished. Only four brood samples had clinical AFB features, but no clear molecular differences between bacterial strains with symptomatic and asymptomatic samples were identified. The reason of this could be the early stage of the disease. According to previous scientific reports AFB progress slower if larvae are infected with ERIC I rather than ERIC II (8, 20). MLVA types differentiated by different loci VNTR allele numbers may appear in the course of adaptation in the host (21).

This study mostly considered the nationwide spatial distribution and variety of MLVA types in the years between 2011 and 2021. The greatest variety of *P. larvae* ERIC I subtypes was detected in bacterial strains collected in 2015, when the majority of samples were collected. It is therefore important to collect a large number of test samples to estimate the dependence of ERIC I subtypes distribution on sampling years for a more objective assessment.

## Conclusions

The infection of a honey bee brood with *P. larvae* was first detected in Lithuania in 2011, and data of 2015 and 2016 showed a widespread distribution of the pathogen. Using the newly adapted MLVA method, the ERIC I genotype was detected in all the *P. larvae* isolates investigated, with the identification of nine different MLVA types of which the most common were MLVA type 1 (44,44%), type 2 (34,26%), and type 3 (12,96%). This method showed good abilities for analyzing bacterial genetic diversity in a restricted area and also usability to trace the geographic distances between origin and mutant strains by comparing how their genetic patterns have changed over time. The present study adds to the understanding of the genetic diversity and geographic distribution of *P. larvae*, facilitating future surveillance of this important honey bee pathogen.

## Data availability statement

The raw data supporting the conclusions of this article will be made available by the authors, without undue reservation.

## Author contributions

All authors listed have made a substantial, direct, and intellectual contribution to the work and approved it for publication.

## Funding

This research project was supported and financed by Support programmes for the Lithuanian beekeeping sector for 2020–2022 programme funds under the measure “Scientific research programs for beekeeping and beekeeping products”.

## Conflict of interest

The authors declare that the research was conducted in the absence of any commercial or financial relationships that could be construed as a potential conflict of interest.

## Publisher's note

All claims expressed in this article are solely those of the authors and do not necessarily represent those of their affiliated organizations, or those of the publisher, the editors and the reviewers. Any product that may be evaluated in this article, or claim that may be made by its manufacturer, is not guaranteed or endorsed by the publisher.
