# Supplementary material for: Molecular diversity of Paenibacillus larvae strains isolated from Lithuanian apiaries
Source: Front Vet Sci. 2022 Aug 22;9:959636. doi: 10.3389/fvets.2022.959636 (PMC9444134; doi:10.3389/fvets.2022.959636)
Supplement: Supplementary file 1 [file Data_Sheet_1.PDF]

Supplementary materials

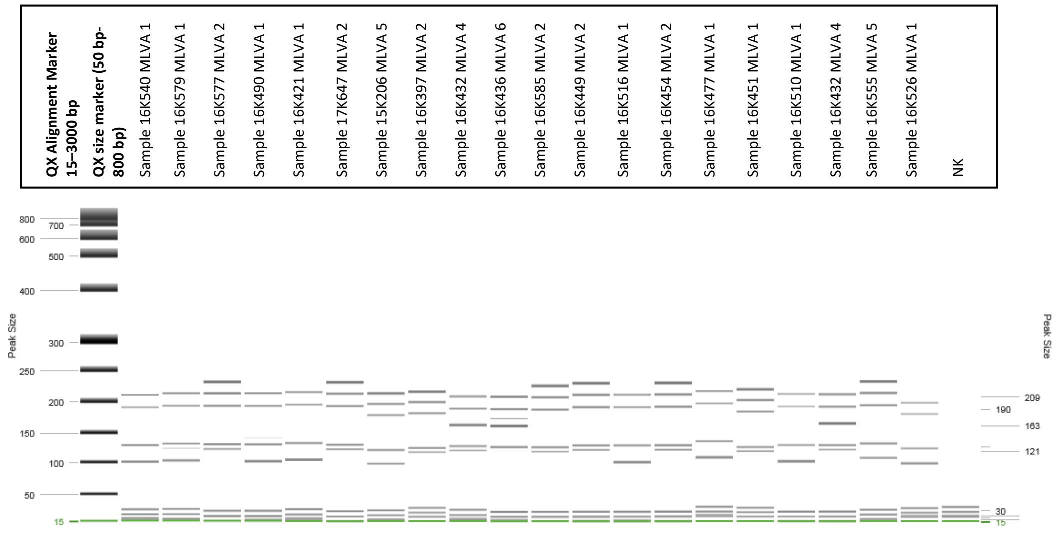

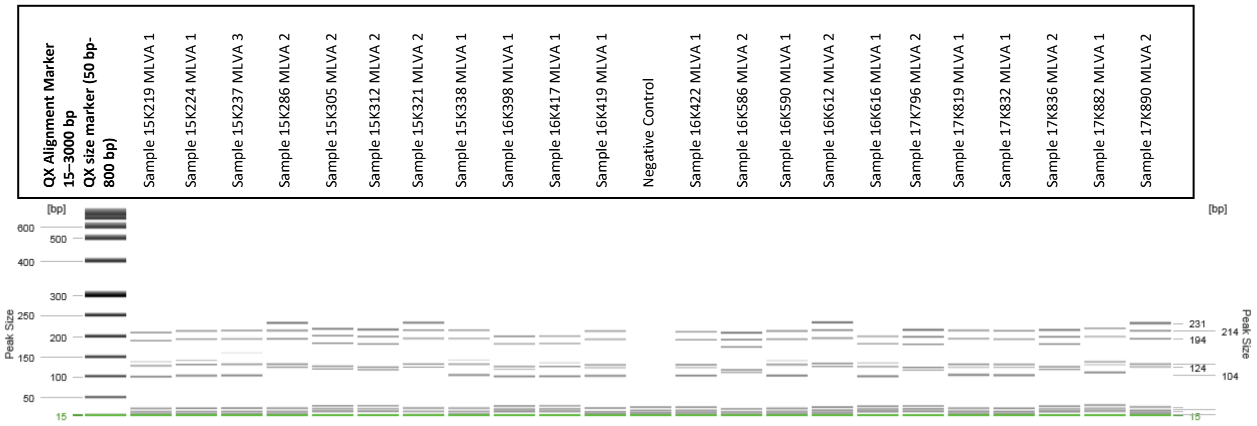

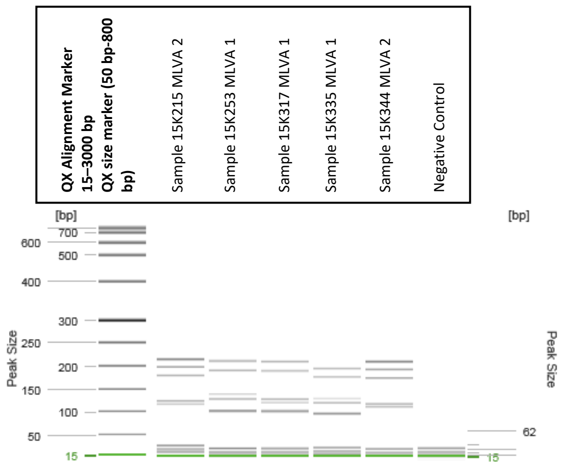

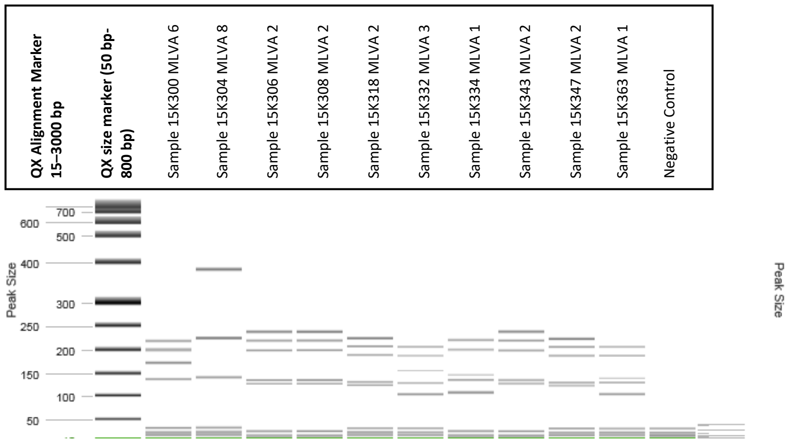
QX Alignment Marker 15–3000 bp
QX size marker (50 bp-800 bp)
Sample 15K300 MLVA 6
Sample 15K304 MLVA 8
Sample 15K306 MLVA 2
Sample 15K308 MLVA 2
Sample 15K318 MLVA 2
Sample 15K332 MLVA 3
Sample 15K334 MLVA 1
Sample 15K343 MLVA 2
Sample 15K347 MLVA 2
Sample 15K363 MLVA 1
Negative Control
Peak Size
700
600
500
400
300
250
200
150
100
50
Peak Size

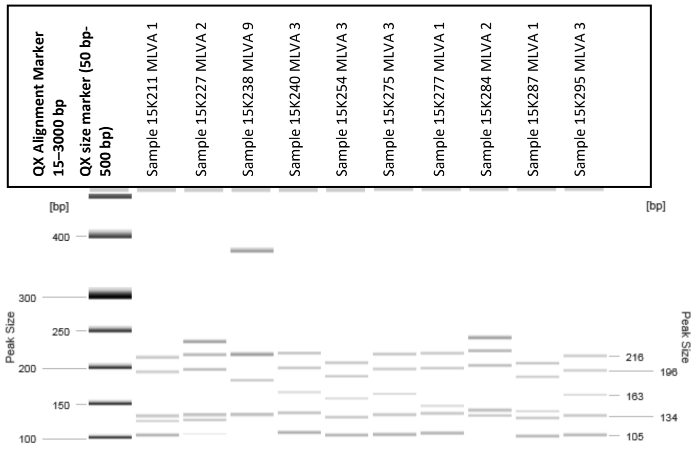
QX Alignment Marker 15–3000 bp
QX size marker (50 bp-500 bp)
Sample 15K211 MLVA 1
Sample 15K227 MLVA 2
Sample 15K238 MLVA 9
Sample 15K240 MLVA 3
Sample 15K254 MLVA 3
Sample 15K275 MLVA 3
Sample 15K277 MLVA 1
Sample 15K284 MLVA 2
Sample 15K287 MLVA 1
Sample 15K295 MLVA 3
[bp]
[bp]
Peak Size
400
300
250
200
150
100
Peak Size
216
196
163
134
105

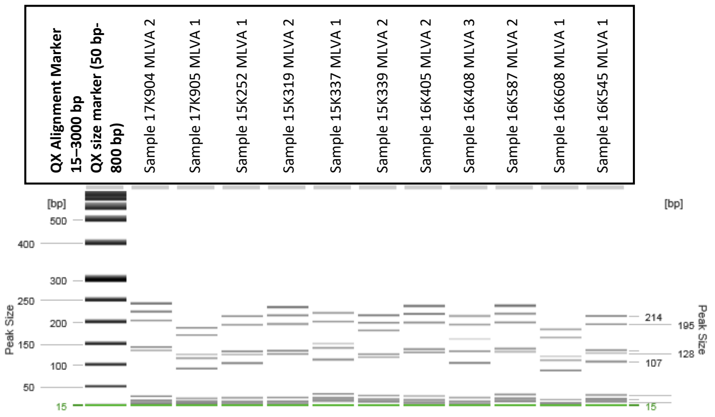
QX Alignment Marker 15–3000 bp
QX size marker (50 bp-800 bp)
Sample 17K904 MLVA 2
Sample 17K905 MLVA 1
Sample 15K252 MLVA 1
Sample 15K319 MLVA 2
Sample 15K337 MLVA 1
Sample 15K339 MLVA 2
Sample 16K405 MLVA 2
Sample 16K408 MLVA 3
Sample 16K587 MLVA 2
Sample 16K608 MLVA 1
Sample 16K545 MLVA 1
[bp]
[bp]
Peak Size
500
400
300
250
200
150
100
50
15
Peak Size
214
195
128
107
15

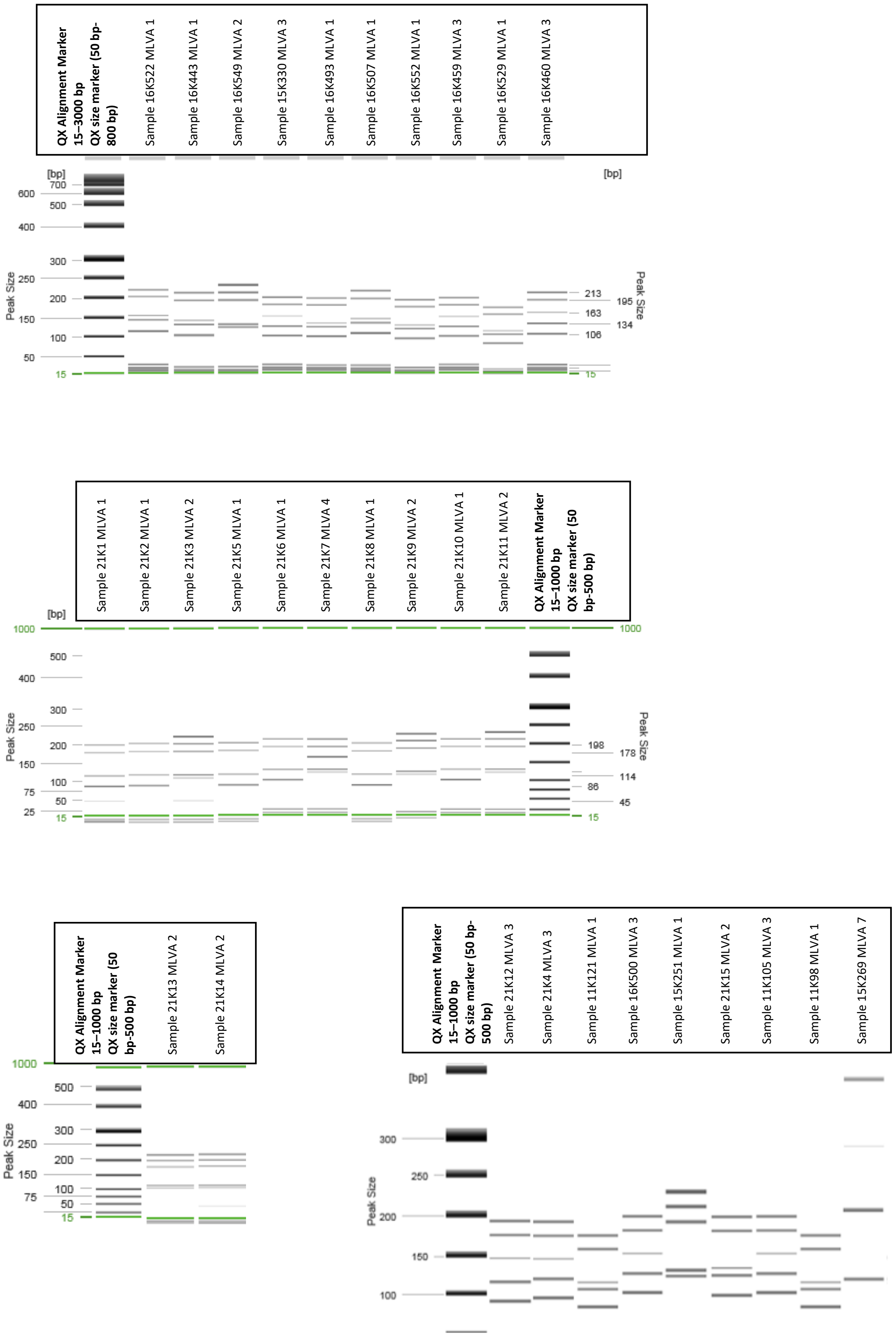

Supplementary Figure S1. QIAxcel capillary electrophoresis images visualise different MLVA types and profiles of each isolate used in this study.
