# Supplementary material for: Molecular diversity of Paenibacillus larvae strains isolated from Lithuanian apiaries
Source: Front Vet Sci. 2022 Aug 22;9:959636. doi: 10.3389/fvets.2022.959636 (PMC9444134; doi:10.3389/fvets.2022.959636)
Supplement: Supplementary file 2 [file Data_Sheet_1.docx]

**Supplementary Table S1.** Calculated theoretical VNTR lengths (bp) of different loci depending on number of tandem repeats (TR)

| VNTR A (TR* 19 bp) | | | | | | | | | | | | | | | |
| --- | --- | --- | --- | --- | --- | --- | --- | --- | --- | --- | --- | --- | --- | --- | --- |
| TR (A) | 1 | **2** | **3** | **4** | **5** | **6** | 7 | 8 | 9 | 10 | **11** | **12** | 13 | 14 | **15** |
| Fragment length, bp | 83 | **102** | **121** | **140** | **159** | **178** | 197 | 216 | 235 | 254 | **273** | **292** | 311 | 330 | **349** |
| VNTR B (TR* 21 bp) | | | | | | | | | | | | | | | |
| TR (B) | 1 | 2 | 3 | 4 | 5 | **6** | **7** | 8 | 9 | 10 | 11 | 12 | 13 | 14 | 15 |
| Fragment length, bp | 86 | 107 | 128 | 149 | 170 | **191** | **212** | 233 | 254 | 275 | 296 | 317 | 338 | 359 | 380 |
| VNTR C (TR* 24 bp) | | | | | | | | | | | | | | | |
| TR (C) | 1 | 2 | **3** | **4** | 5 | 6 | 7 | 8 | 9 | 10 | 11 | 12 | 13 | 14 | 15 |
| Fragment length, bp | 151 | 175 | **199** | **223** | 247 | 271 | 295 | 319 | 343 | 367 | 391 | 415 | 439 | 463 | 487 |
| VNTR D (TR* 24 bp) | | | | | | | | | | | | | | | |
| TR (D) | 1 | 2 | **3** | 4 | 5 | 6 | 7 | 8 | 9 | 10 | 11 | 12 | 13 | 14 | 15 |
| Fragment length, bp | 77 | 101 | **125** | 149 | 173 | 197 | 221 | 245 | 269 | 293 | 317 | 341 | 365 | 389 | 413 |
| VNTR E (TR* 68 bp) | | | | | | | | | | | | | | | |
| TR (E) | **1** | **2** | 3 | **4** | 5 | 6 | 7 | 8 | 9 | 10 | 11 | 12 | 13 | 14 | 15 |
| Fragment length, bp | **188** | **256** | 324 | **392** | 460 | 528 | 596 | 664 | 732 | 800 | 868 | 936 | 1004 | 1072 | 1140 |

**Supplementary Table S2**. Different loci allele tandem repeats (TR) theorical (expected) sizes and QIAcxel measured sizes comparison

| PCR | Locus | Allele (TR) | Expected size, bp | QIAxel | |
| --- | --- | --- | --- | --- | --- |
|  |  |  |  | Measured size, bp | A±SD^b^, bp |
| Multiplex/singleplex^a^ | VNTR A | 2 | 102 | 99-104 | 101.4±2.41 |
|  |  | 3 | 121 | 117-147 | 132.15±8.36 |
|  |  | 4 | 140 |  |  |
|  |  | 5 | 159 | 152-169 | 159.21±5.10 |
|  |  | 6 | 178 |  |  |
|  |  | 11 | 273 | 264-270 | 268.5±3.0 |
|  |  | 13 | 311 | 286-299 | 294±7.0 |
|  |  | 15 | 349 | 336-350 | 345.33±8.08 |
| Multiplex/singleplex^a^ | VNTR B | 6 | 191 | 174-205 | 190.09±7.95 |
|  |  | 7 | 212 | 193-210 | 203.62±8.88 |
| Multiplex/singleplex^a^ | VNTR C | 3 | 199 | 183-224 | 209.11±8.69 |
|  |  | 4 | 223 |  |  |
| Multiplex/singleplex^a^ | VNTR D | 3 | 125 | 104-138 | 124.81±8.07 |
| Multiplex/singleplex^a^ | VNTR E | 0 | - | 83-110 | 99.88±6.47 |
|  |  | 1 | 188 | 163-173 | 166.4±5.56 |
|  |  | 2 | 256 | 227-242 | 226.45±10.46 |
|  |  | 4 | 392 | 364-388 | 374.42±7.15 |

^a^ *VNTR lengths were amplified by using both PCR methods for confirmation.*

^b^ *Arithmetic average (A) ± standard deviation (SD) of the observed sizes.*

**Supplementary Table S3.** Different MLVA types divided according VNTR A, VNTR B, VNTR C, VNTR D and VNTR E lengths of each isolate

| Years | Name of isolate | VNTR (A, B, C, D, E) average length, bp* | | | | | | | | | | MLVA type | ERIC genotype |
| --- | --- | --- | --- | --- | --- | --- | --- | --- | --- | --- | --- | --- | --- |
| 2011 | **11K121** | (E) 94 | (D) 106 | (A) 118 |  |  | (B) 176 | (C) 194 |  |  |  | **1** | **I** |
|  | **11K98** | (E) 94 | (D) 106 | (A) 118 |  |  | (B) 176 | (C) 194 |  |  |  | **1** | **I** |
| 2015 | **15K253** | (E) 103 | (D)130 | (A) 141 |  |  | (B) 192 | (C) 212 |  |  |  | **1** | **I** |
|  | **15K317** | (E) 102 | (D) 122 | (A) 130 |  |  | (B) 192 | (C) 211 |  |  |  | **1** | **I** |
|  | **15K335** | (E) 100 | (D) 124 | (A) 133 |  |  | (B) 180 | (C)198 |  |  |  | **1** | **I** |
|  | **15K252** | (E) 104 | (D) 125 | (A) 132 |  |  | (B) 194 | (C) 213 |  |  |  | **1** | **I** |
|  | **15K337** | (E) 98 | (D) 107 | (A) 120 |  |  | (B)183 | (C)195 |  |  |  | **1** | **I** |
|  | **15K211** | (E)104 | (D) 124 | (A) 132 |  |  | (B) 194 | (C) 214 |  |  |  | **1** | **I** |
|  | **15K277** | (E) 107 | (D) 133 | (A) 148 |  |  | (B) 192 | (C) 210 |  |  |  | **1** | **I** |
|  | **15K287** | (E) 103 | (D) 130 | (A) 140 |  |  | (B) 188 | (C) 206 |  |  |  | **1** | **I** |
|  | **15K334** | (E) 107 | (D)132 | (A) 144 |  |  | (B) 200 | (C) 221 |  |  |  | **1** | **I** |
|  | **15K363** | (E) 103 | (D) 129 | (A) 139 |  |  | (B) 188 | (C)206 |  |  |  | **1** | **I** |
|  | **15K219** | (E) 99 | (D) 127 | (A) 138 |  |  | (B) 190 | (C) 209 |  |  |  | **1** | **I** |
|  | **15K224** | (E )101 | (D) 130 | (A) 141 |  |  | (B) 193 | (C) 213 |  |  |  | **1** | **I** |
|  | **15K338** | (E)103 | (D) 131 | (A) 142 |  |  | (B) 195 | (C) 215 |  |  |  | **1** | **I** |
|  | **15K251** | (E) 98 | (D)110 | (A) 133 |  |  | (B)178 | (C) 203 |  |  |  | **1** | **I** |
| 2016 | **16K608** | (E) 87 | (D) 111 | (A) 120 |  |  | (B) 176 | (C)186 |  |  |  | **1** | **I** |
|  | **16K545** | (E) 107 | (D) 128 | (A) 135 |  |  | (B) 195 | (C) 214 |  |  |  | **1** | **I** |
|  | **16K522** | (E) 110 | (D) 134 | (A) 148 |  |  | (B)201 | (C) 219 |  |  |  | **1** | **I** |
|  | **16K443** | (E) 103 | (D) 131 | (A) 142 |  |  | (B) 193 | (C) 212 |  |  |  | **1** | **I** |
|  | **16K493** | (E) 100 | (D) 125 | (A) 135 |  |  | (B) 182 | (C)199 |  |  |  | **1** | **I** |
|  | **16K507** | (E) 108 | (D) 132 | (A) 144 |  |  | (B) 198 | (C) 218 |  |  |  | **1** | **I** |
|  | **16K552** | (E) 94 | (D) 120 | (A) 130 |  |  | (B) 179 | (C)199 |  |  |  | **1** | **I** |
|  | **16K529** | (E)83 | (D) 106 | (A) 118 |  |  | (B) 176 | (C)188 |  |  |  | **1** | **I** |
|  | **16K398** | (E) 100 | (D) 118 | (A) 125 |  |  | (B) 182 | (C) 201 |  |  |  | **1** | **I** |
|  | **16K417** | (E) 100 | (D) 125 | (A)135 |  |  | (B)183 | (C) 201 |  |  |  | **1** | **I** |
|  | **16K419** | (E) 101 | (D) 122 | (A) 130 |  |  | (B) 193 | (C) 213 |  |  |  | **1** | **I** |
|  | **16K422** | (E) 102 | (D) 123 | (A) 130 |  |  | (B) 192 | (C) 211 |  |  |  | **1** | **I** |
|  | **16K590** | (E) 102 | (D)130 | (A) 141 |  |  | (B) 193 | (C) 213 |  |  |  | **1** | **I** |
|  | **16K616** | (E) 100 | (D) 125 | (A) 135 |  |  | (B) 182 | (C)200 |  |  |  | **1** | **I** |
|  | **16K540** | (E) 101 | (D) 129 | (A) 145 |  |  | (B) 191 | (C)211 |  |  |  | **1** | **I** |
|  | **16K579** | (E)103 | (D) 124 | (A) 141 |  |  | (B) 194 | (C)214 |  |  |  | **1** | **I** |
|  | **16K490** | (E) 101 | (D)130 | (A) 141 |  |  | (B) 193 | (C)213 |  |  |  | **1** | **I** |
|  | **16K421** | (E)105 | (D) 133 | (A) 149 |  |  | (B) 196 | (C)216 |  |  |  | **1** | **I** |
|  | **16K516** | (E)100 | (D) 129 | (A) 141 |  |  | (B) 192 | (C)212 |  |  |  | **1** | **I** |
|  | **16K477** | (E)109 | (D)137 | (A) 147 |  |  | (B) 198 | (C)218 |  |  |  | **1** | **I** |
|  | **16K510** | (E)102 | (D) 130 | (A) 141 |  |  | (B) 193 | (C)213 |  |  |  | **1** | **I** |
|  | **16K526** | (E) 99 | (D) 124 | (A) 134 |  |  | (B) 181 | (C)199 |  |  |  | **1** | **I** |
| 2017 | **17K905** | (E) 91 | (D) 116 | (A) 125 |  |  | (B) 178 | (C)187 |  |  |  | **1** | **I** |
|  | **17K819** | (E) 103 | (D) 123 | (A) 131 |  |  | (B)199 | (C) 214 |  |  |  | **1** | **I** |
|  | **17K832** | (E) 103 | (D) 123 | (A) 130 |  |  | (B) 199 | (C) 214 |  |  |  | **1** | **I** |
|  | **17K882** | (E) 110 | (D) 130 | (A) 138 |  |  | (B) 199 | (C) 219 |  |  |  | **1** | **I** |
| 2021 | **21K1** | (E) 86 | (D) 114 | (A)125 |  |  | (B)178 | (C) 198 |  |  |  | **1** | **I** |
|  | **21K2** | (E) 88 | (D) 117 | (A)128 |  |  | (B)182 | (C) 203 |  |  |  | **1** | **I** |
|  | **21K5** | (E) 88 | (D) 114 | (A)128 |  |  | (B)182 | (C) 203 |  |  |  | **1** | **I** |
|  | **21K6** | (E) 101 | (D) 131 | (A)141 |  |  | (B)193 | (C) 213 |  |  |  | **1** | **I** |
|  | **21K8** | (E) 88 | (D) 109 | (A)117 |  |  | (B)181 | (C) 202 |  |  |  | **1** | **I** |
|  | **21K10** | (E) 102 | (D)131 | (A)142 |  |  | (B)193 | (C) 213 |  |  |  | **1** | **I** |
| 2015 | **15K215** |  | (D) 115 | (A) 122 |  |  | (B) 185 | (C) 200 | (E) 227 |  |  | **2** | **I** |
|  | **15K344** |  | (D) 112 | (A) 120 |  |  | (B) 176 | (C)194 | (E) 235 |  |  | **2** | **I** |
|  | **15K319** |  | (D) 126 | (A) 134 |  |  | (B) 196 | (C) 216 | (E) 234 |  |  | **2** | **I** |
|  | **15K339** |  | (D) 118 | (A) 125 |  |  | (B)181 | (C)199 | (E) 215 |  |  | **2** | **I** |
|  | **15K227** |  | (D) 126 | (A) 134 |  |  | (B) 197 | (C) 218 | (E) 235 |  |  | **2** | **I** |
|  | **15K284** |  | (D) 134 | (A) 141 |  |  | (B) 203 | (C) 223 | (E) 241 |  |  | **2** | **I** |
|  | **15K306** |  | (D) 127 | (A) 135 |  |  | (B) 199 | (C) 219 | (E) 237 |  |  | **2** | **I** |
|  | **15K308** |  | (D) 127 | (A) 135 |  |  | (B) 199 | (C) 219 | (E) 237 |  |  | **2** | **I** |
|  | **15K318** |  | (D) 124 | (A) 131 |  |  | (B) 189 | (C)208 | (E) 224 |  |  | **2** | **I** |
|  | **15K343** |  | (D) 127 | (A) 135 |  |  | (B) 199 | (C) 219 | (E) 237 |  |  | **2** | **I** |
|  | **15K347** |  | (D) 122 | (A) 129 |  |  | (B) 188 | (C)206 | (E) 223 |  |  | **2** | **I** |
|  | **15K286** |  | (D) 123 | (A) 131 |  |  | (B) 195 | (C) 214 | (E) 232 |  |  | **2** | **I** |
|  | **15K305** |  | (D) 119 | (A) 126 |  |  | (B) 184 | (C) 202 | (E) 218 |  |  | **2** | **I** |
|  | **15K312** |  | (D) 117 | (A) 124 |  |  | (B) 182 | (C) 200 | (E) 216 |  |  | **2** | **I** |
|  | **15K321** |  | (D) 124 | (A) 131 |  |  | (B) 195 | (C) 215 | (E) 233 |  |  | **2** | **I** |
| 2016 | **16K405** |  | (D) 129 | (A) 137 |  |  | (B) 199 | (C) 219 | (E) 237 |  |  | **2** | **I** |
|  | **16K587** |  | (D) 131 | (A) 139 |  |  | (B) 199 | (C)219 | (E) 238 |  |  | **2** | **I** |
|  | **16K549** |  | (D) 124 | (A) 132 |  |  | (B) 194 | (C)213 | (E) 232 |  |  | **2** | **I** |
|  | **16K586** |  | (D) 110 | (A) 117 |  |  | (B) 174 | (C) 192 | (E) 209 |  |  | **2** | **I** |
|  | **16K612** |  | (D) 125 | (A) 133 |  |  | (B) 196 | (C) 215 | (E) 233 |  |  | **2** | **I** |
|  | **16K577** |  | (D) 122 | (A)130 |  |  | (B) 193 | (C)213 | (E) 232 |  |  | **2** | **I** |
|  | **16K397** |  | (D) 118 | (A) 125 |  |  | (B) 182 | (C) 200 | (E)217 |  |  | **2** | **I** |
|  | **16K585** |  | (D)119 | (A) 126 |  |  | (B) 188 | (C)208 | (E) 226 |  |  | **2** | **I** |
|  | **16K449** |  | (D) 122 | (A)129 |  |  | (B) 192 | (C)212 | (E) 230 |  |  | **2** | **I** |
|  | **16K454** |  | (D) 122 | (A) 130 |  |  | (B) 193 | (C)213 | (E) 231 |  |  | **2** | **I** |
|  | **16K451** |  | (D) 119 | (A) 127 |  |  | (B) 185 | (C)185 | (E) 203 |  |  | **2** | **I** |
| 2017 | **17K904** |  | (D)134 | (A)143 |  |  | (B) 203 | (C) 224 | (E) 242 |  |  | **2** | **I** |
|  | **17K796** |  | (D) 116 | (A) 123 |  |  | (B) 181 | (C)199 | (E)205 |  |  | **2** | **I** |
|  | **17K836** |  | (D) 118 | (A) 124 |  |  | (B) 181 | (C) 199 | (E) 205 |  |  | **2** | **I** |
|  | **17K890** |  | (D) 124 | (A) 132 |  |  | (B) 199 | (C) 214 | (E) 231 |  |  | **2** | **I** |
|  | **17K647** |  | (D) 123 | (A) 130 |  |  | (B) 194 | (C)214 | (E) 232 |  |  | **2** | **I** |
| 2021 | **21K3** |  | (D) 109 | (A) 117 |  |  | (B) 181 | (C) 202 | (E) 221 |  |  | **2** | **I** |
|  | **21K9** |  | (D) 117 | (A) 125 |  |  | (B) 176 | (C) 188 | (E) 208 |  |  | **2** | **I** |
|  | **21K11** |  | (D) 123 | (A) 131 |  |  | (B) 179 | (C) 193 | (E) 213 |  |  | **2** | **I** |
|  | **21K13** |  | (D) 108 | (A) 117 |  |  | (B) 180 | (C) 201 | (E) 220 |  |  | **2** | **I** |
|  | **21K14** |  | (D) 110 | (A) 118 |  |  | (B) 183 | (C) 204 | (E) 223 |  |  | **2** | **I** |
|  | **21K15** |  | (D) 125 | (A)134 |  |  | (B) 194 | (C) 204 | (E) 231 |  |  | **2** | **I** |
| 2011 | **11K105** | (E) 95 | (D) 119 |  | (A) 152 |  | (B) 178 | (C) 194 |  |  |  | **3** | **I** |
| 2015 | **15K330** | (E) 101 | (D) 127 |  | (A) 154 |  | (B) 183 | (C) 201 |  |  |  | **3** | **I** |
|  | **15K240** | (E) 107 | (D) 134 |  | (A) 166 |  | (B) 200 | (C)220 |  |  |  | **3** | **I** |
|  | **15K254** | (E) 103 | (D) 130 |  | (A) 157 |  | (B) 188 | (C) 207 |  |  |  | **3** | **I** |
|  | **15K275** | (E)105 | (D) 135 |  | (A) 164 |  | (B) 198 | (C)219 |  |  |  | **3** | **I** |
|  | **15K295** | (E) 105 | (D)134 |  | (A) 163 |  | (B)196 | (C) 216 |  |  |  | **3** | **I** |
|  | **15K332** | (E) 103 | (D)129 |  | (A) 156 |  | (B) 188 | (C)207 |  |  |  | **3** | **I** |
|  | **15K237** | (E) 102 | (D) 131 |  | (A) 160 |  | (B) 194 | (C) 214 |  |  |  | **3** | **I** |
| 2016 | **16K408** | (E) 102 | (D) 133 |  | (A)162 |  | (B)194 | (C) 214 |  |  |  | **3** | **I** |
|  | **16K500** | (E) 86 | (D)115 |  | (A) 159 |  | (B) 178 | (C) 210 |  |  |  | **3** | **I** |
|  | **16K459** | (E)100 | (D) 126 |  | (A) 153 |  | (B)182 | (C)200 |  |  |  | **3** | **I** |
|  | **16K460** | (E) 106 | (D) 134 |  | (A) 163 |  | (B) 195 | (C)213 |  |  |  | **3** | **I** |
| 2021 | **21K4** | (E) 89 | (D) 120 |  | (A) 153 |  | (B) 184 | (C) 204 |  |  |  | **3** | **I** |
|  | **21K12** | (E) 85 | (D) 114 |  | (A) 167 |  | (B) 181 | (C) 197 |  |  |  | **3** | **I** |
| 2016 | **16K432** |  | (D) 125 | (A) 132 | (E) 167 |  | (B) 194 | (C) 213 |  |  |  | **4** | **I** |
| 2021 | **21K7** |  | (D) 126 | (A) 134 | (E) 167 |  | (B) 195 | (C) 215 |  |  |  | **4** | **I** |
| 2015 | **15K206** | (A) 100 | (D) 126 |  |  |  | (B) 185 | (C) 205 | (E) 225 |  |  | **5** | **I** |
| 2016 | **16K555** | (A)104 | (D)133 |  |  |  | (B) 195 | (C)215 | (E) 233 |  |  | **5** | **I** |
| 2015 | **15K300** |  | (D) 124 |  | (E) 155 | (A) 172 | (B)195 | (C)210 |  |  |  | **6** | **I** |
| 2016 | **16K436** |  | (D) 133 |  | (E)167 | (A) 180 | (B) 195 | (C) 215 |  |  |  | **6** | **I** |
| 2015 | **15K269** |  | (D) 136 |  |  |  | (B) 210 | (C) 218 |  | (A) 298 | (E) 378 | **7** | **I** |
| 2015 | **15K304** |  | (D) 130 |  |  |  | (B) 208 | (C) 213 |  | (A) 350 | (E) 368 | **8** | **I** |
| 2015 | **15K238** |  | (D) 130 |  |  | (A) 177 | (B) 208 | (C) 214 |  |  | (E) 367 | **9** | **I** |
|  | ***p.larvae* ATCC 9545** |  | (D) 127 |  |  |  | (B) 208 | (C) 211 |  | (A) 269 | (E) 371 |  | **I** |

**Average number of tandem repeats (bp) after several repetitions of each locus*
